# The Use of Taxis in Reducing Herniæ

**Published:** 1899-05-06

**Authors:** W. McAdam Eccles

**Affiliations:** Assistant Surgeon to the City of London Truss Society, to the West London Hospital, &c.


### Hospital Clinics and Medical Progress.

### THE USE OF TAXIS IN REDUCING HERNIÆ.

By W. McAdam Eccles, M.S.Lond., F.R.C.S.Eng., Assistant Surgeon to the City of London Truss Society, to the West London Hospital, &c.

Taxis may be defined as the external manipulation of a hernial swelling with a view to the reduction of the contents of the sac. It may be employed upon a reducible, a simple irreducible, and a strangulated hernia, but the method with which it is carried out under these different conditions varies considerably. Taxis when required for a hernia which is ordinarily reducible is not much more than a simple manipulation, which in many cases the patient is able to perform without assistance. He assumes the horizontal posture, the hernia is not spontaneously reducible, and then, often in a manner which is peculiar to himself and to his hernia, he employs pressure and movement, usually with both his hands. In this way the contents of the sac will in the end return into the abdominal cavity. I have many times been struck with the ease with which some patients can of themselves bring about the reduction of their hernial protrusions, even where the action of the surgeon has failed. It is rare for a patient to use such pressure that he will experience discomfort, though at times doubtless he has more difficulty than at others in producing reduction.

A simple irreducible hernia is one in which the contents of the sac are merely irreducible, and, at the same time, there is no interference with the circulation of blood through the bowel wall. It is important to recognise that simple irreducibility is but a transitory condition in the majority of instances. A hernia which is irreducible to-day may be wholly reducible to-morrow; but, generally speaking, the longer a hernia has been irreducible, the less likelihood there is that reduction will be effected. Taxis, when employed for a simple irreducible hernia, can be used in a manner altogether different to that which would be justifiable in the case of strangulated hernia. In the former, the contents of the sac, whether gut or omentum, are in a normal condition, with a satisfactory return of their venous blood. In the latter, the intestine is subjected to constriction, with the effect that the bowel is in by no means a normal state. If the example of a simple irreducible right scrotal hernia, perhaps the most common of all irreducible herniæ, be taken, taxis should be carried out in the following manner.

The patient is made to assume the dorsal posture, on a couch which is somewhat low, so that the surgeon can stoop over him. His buttocks may be raised, though such a position is not essential, and possibly does not aid much the return of the viscera. With regard to the lie of the right lower limb, there is some difference of opinion. Many consider that it is best to have the extremity flexed at both hip and knee, and at the same time adducted and internally rotated, so that the hernial apertures, and the superficial ring particularly, may be relaxed. It is thought hereby that the return of the contents of the sac will be greatly facilitated. To my mind, however, this position is altogether a mistaken one, for if one desires to reduce the viscera, it is much sounder practice to have the rings taut, so that they do not themselves give before the force which is being applied to the sac. Again, the position of the limb in adduction and flexion almost of necessity effectually prevents a satisfactory manipulation of the hernial protrusion in the manner about to be described. I think, therefore, that the proper posture in which the limb should rest is one of extension, with slight abduction and external rotation, and in my experience this is the position that most surgeons allow it to assume while attempting reduction by taxis.

The surgeon himself stands on the right side of the couch, looking towards the patient's face, when the hernia is but small. He then applies his right hand to the swelling, while his left should lie over the inguinal canal, with the forefinger and thumb making as it were a groove into which the contents of the sac can be pushed. At the same time these digits tend to keep the neck of the sac taut and drawn downwards. If, on the other hand, the hernia is of some considerable magnitude, then the surgeon should stand looking towards the patient's feet, and should grasp the whole of the hernial protrusion in his two hands in such a manner that his fingers embrace the bulk of the swelling, while his thumbs are directed upwards and outwards in the line of the inguinal canal.

It is always advisable to remember that the part of the contents which descended the latest into the sac, and which usually lies at the posterior part of the sac, is the first to be reduced. Thus the manipulation with the fingers in this second position resolves itself into a species of a revolution, as the surgeon's fingers should exercise most pressure primarily on the hinder part of the sac, then on the lower, and finally on the anterior portion. Intestine is generally behind, and this commonly slips back with its characteristic gurgle, and with comparatively little difficulty. The omentum lies in front, and often is more resistant to the effect of pressure. Care must be taken to prevent the crowding up of this structure over the superficial ring. This can be readily prevented by the action of the thumbs in the position that they occupy over the ring and the canal. Sometimes the omentum is pushed up into the canal, but does not pass into the abdominal cavity through the deep ring. If this should happen, and it is easy of recognition by the continuance of the swelling, no further attempt at reduction should be made until the omentum has been again drawn down by the fingers, and the whole proceeding recommenced.

In very large, tense scrotal or labial herniæ it is well for the surgeon to place his hands in yet another posture, one hand on either side of the hernial protrusion, and the wrists in the position that the thumbs occupied above, that is, over the neck of the sac. The hands should endeavour, with the help of the wrists, to draw the sac downwards and to keep it taut, while the fingers particularly exert the necessary pressure on the contents of the sac.

A very considerable amount of force may be employed in the case of a simple irreducible hernia; in fact, all that the surgeon can use with his hands and the weight that he can throw into his actions. This, it must be again pointed out, would be wholly unjustifiable in the instance of a strangulated hernia.

If the contents of the sac are beginning to return to their natural habitat the patient usually experiences some degree of pain, and loudly in some cases calls for a cessation of manipulation. It is now that the surgeon by continuing his efforts may have them crowned with success. The movement of the contents of a sac, whether it be in an upward or a downward direction, seems to be almost invariably accompanied by pain; but a patient may lie oblivious to the effects of the pressure applied so long as that pressure does not bring about any change of position of the contents of the sac. Taxis is usually efficacious in recent irreducibility, in children, and in inguinal herniæ more than in femoral herniæ.

When the surgeon has to deal with a case where strangulation is in evidence quite another condition exists than that of a simple irreducible hernia, and this is to be clearly recognised, even though at the risk of repeating the statement. It is doubtful whether the majority of cases of strangulated herniæ would not be better dealt with by subjecting them forthwith to the operation of herniotomy without any attempt at taxis. But there are undoubtedly some cases in which taxis should be at any rate the first method of treatment adopted. Such is the case if the hernia be that of a young child, a feeble old person, or if it be a very large protrusion.

It is not easy to get efficient pressure upon the contents of the sac of a strangulated hernia, owing to the presence of fluid in the sac. It is, moreover, difficult to cause this fluid to be reduced into the abdomen, and it is doubtful whether it is always a safe matter to bring about its return, for it may have become highly septic from bacteria which have transgressed from the cavity of the intestine.

The same general posture of patient and surgeon is assumed as in the case of the non-strangulated protrusion. The amount of force to be used must be very much less than that which is justifiable in the case of a simple irreducible hernia. Also, this pressure must not be long continued, for there are very grave dangers in it. It were surely safer to let the patient run the risk of an operation with undamaged bowel, at least so far as the taxis is responsible, than to put the herniotomy off until severe injury has been inflicted upon the intestine, as the result of the energetic attempts at reduction.

The whole of the contents of the sac must be completely reduced when taxis is employed, for it is not safe to leave even a small portion behind, for this may perhaps be the very part that is still nipped. If any swelling still remains after reduction, or apparent reduction, then an operation ought to be undertaken immediately.

Adjuncts to taxis in strangulated herniæ rather belie their name, for it is rare that they really help to bring about the return of the contents. If the patient is given a hot bath, taxis should certainly not be begun while the sufferer is still in the water, but he should be removed from the bath and placed between hot blankets for at least ten minutes before the pressure is applied. Again, if opium is administered it is well to allow it to have its full effect before the attempt to return the bowel is made. It will thus be seen that these so-called adjuncts tend in the main to delay, and do not markedly aid the liberation of the imprisoned intestine.

